# Nutritional Status, School Time, and Eating Patterns in Children From 7 to 10 Years: Quantitative Evaluation of Macro and Micronutrients in the School Diet

**DOI:** 10.1002/fsn3.71719

**Published:** 2026-04-15

**Authors:** Daniel Ramos Olcerenko, Bruna da Silva Miranda, Erica Vanessa Batista dos Santos, Lucas Melo Neves, Patrícia Colombo‐Souza

**Affiliations:** ^1^ Postgraduate Program in Health Sciences Santo Amaro University ‐ Unisa São Paulo Brazil; ^2^ Nutrition Course Santo Amaro University ‐ Unisa São Paulo Brazil; ^3^ Physical Activity, Sport and Mental Health Laboratory (LAFESAM), Department of Physical Education, Institute of Biosciences São Paulo State University (UNESP) Rio Claro Brazil

**Keywords:** child nutrition, macronutrients, nutritional status, schoolchildren, sodium

## Abstract

To quantitatively evaluate the intake of key macronutrients and sodium in the diet of children aged 7–10 years and their relationship with nutritional status and school schedule, this cross‐sectional study analyzed dietary data from guardian questionnaires using the Yazio, Food Converter, and Web Diet platforms. Nutrient intake was compared against guidelines, and statistical analyses included Chi‑squared, Mann–Whitney, and Kruskal–Wallis tests (*p* < 0.05). Data were collected from private schools in Cotia, São Paulo, Brazil, between January and August 2023. The sample comprised 60 children (27 girls, 33 boys; mean age 8.6 years), with 70% enrolled part‑time. Overall, 58.3% were eutrophic, while 41.7% had an altered nutritional status. Paradoxically, despite a high prevalence of overweight/obesity (26.7%), carbohydrate and fat intake were below recommendations for all children, suggesting poor diet quality. In contrast, 55% met water intake guidelines, while 48.3% exceeded sodium limits. Significant differences in intake were found across nutritional status categories (*p* < 0.001). Full‑time schooling was associated with higher carbohydrate intake (*p* = 0.018) and a trend toward higher sodium consumption (*p* = 0.073), but not with nutritional status (*p* = 0.298). The study reveals a double burden of poor diet quality, with full‑time schooling linked to higher intake of critical nutrients. This underscores the need for policies that regulate the school food environment, particularly in full‑time settings, and promote food education.

## Graphical abstract

Nutritional status, school time, and eating patterns in children aged 7–10 years: quantitative evaluation of macro‐ and micronutrient intake in the school diet.

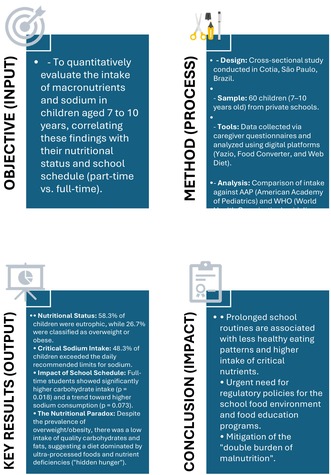

## 1 Introduction

The transition from childhood to adulthood, driven by accelerated urbanization and socioeconomic changes, has reconfigured the nutritional habits of children on a global scale (Damassini and Bruch‐Bertani 2023). In the Brazilian context, where more than 85% of the population resides in urban areas, there is a progressive substitution of in‐natura foods by industrialized products rich in sodium, free sugars, and saturated fat (Cainelli et al. 2021; Mescoloto et al. 2024; Pérez‐Escamilla 2023). These patterns, associated with the busy routine of families in which both parents work away from home, favor the consumption of ready meals and processed or ultra‐processed foods, which correspond to about 85% of children's current food ingestion (Martí Del Moral et al. 2021). This scenario is of concern because it is directly linked to the increase in overweight, obesity, and chronic non‐communicable diseases (CNCD) in schoolchildren, such as diabetes and arterial hypertension, conditions that were previously predominantly adult (Rauber et al. 2021; Chang et al. 2023).

The school environment is a critical point for intervention, as children spend a significant portion of their day there. However, the school's influence is ambivalent. On the one hand, it can be a place that promotes healthy eating; on the other hand, it can expose children to unhealthy options. This dynamic is a global challenge; studies from South Africa highlight that school tuck shops and street vendors around schools predominantly sell non‐nutritious foods, such as chips, cakes, and sweets. The practice of children using pocket money to purchase these items is a significant contributor to poor dietary choices (Phetla et al. 2024). Similarly, research from Russia reports high consumption of ultra‐processed foods and sweets among students, while in Saudi Arabia, skipping meals—particularly breakfast—is a common and concerning practice linked to obesity (Podchinenova et al. 2023; Güven and Öncü 2022).

The Pan American Health Organization (PAHO) emphasizes that childhood is a critical period for the consolidation of eating habits, with repercussions throughout life (Organização Pan‐Americana da Saúde 2005). Children between 7 and 10 years of age, a phase marked by accelerated growth and cognitive development, require specific nutrients in balanced quantities (Ministério da Saúde 2018). However, studies indicate that the diet of this age group often exceeds the recommended limits for sodium (WHO recommendation: < 2 g/day) and free sugars (< 10% of total calories). At the same time, it has deficits in essential micronutrients, such as iron and vitamins (Organización Panamericana de la Salud 2021; World Health Organization [WHO] 2015). In addition, inadequate water consumption, which has a daily recommendation of between 1.7 and 2.4 L, depending on weight, height, and physical activity, aggravates metabolic and cognitive risks (Armstrong et al. 2024).

In Brazil, this scenario is further complicated by the type of school and the school schedule. While Brazilian public schools follow the guidelines of the National School Feeding Program (PNAE), which prioritizes fresh food and limits processed foods, private institutions do not always adopt similar protocols (Nero et al. 2023). This regulatory disparity creates an environment of greater risk in private schools, a concern corroborated by international evidence on the role of unregulated food sales in schools (Phetla et al. 2024). Interestingly, children in private schools, whether full‐time (morning and afternoon) or part‐time (morning or afternoon), show high consumption of processed and ultra‐processed foods (Henriques et al. 2021). This raises a crucial question: Does a longer school day (full‐time) increase exposure to unhealthy foods at school, or do part‐time students compensate with a higher intake of these foods at home? Investigating the correlation between school schedule and nutritional status is therefore essential to understand these contextual influences.

In addition to the constitution of processed and ultra‐processed foods, early exposure to intense flavors (such as salt, sugar, and fat) also plays a central role in forming food preferences. Evidence suggests that the introduction of processed foods before 2 years of age is associated with greater acceptance of these products in childhood and adolescence (Brito and Andrade 2022). This cycle is perpetuated by the food industry, which uses additives to increase palatability, creating a disconnection between physiological needs and dietary choices (Jachimowicz‐Rogowska and Winiarska‐Mieczan 2023; Ministério da Saúde 2022). It is essential to highlight that within the scope of public policies, global targets such as the 30% reduction in salt consumption by 2025 (WHO) and the eradication of trans‐industrial fats by 2023 (PAHO) underscore the urgency of monitoring dietary patterns in vulnerable populations, including schoolchildren (WHO 2015, 2023).

The majority of research on child nutritional intake in Brazil focuses on public school populations, often associated with socioeconomic vulnerabilities. This study focuses on children from 7 to 10 years enrolled in private schools in the city of Cotia (São Paulo), a group that represents 18% of national enrollments in elementary school and whose socioeconomic profile, although heterogeneous, is marked by greater access to financial resources. The lack of evidence on the eating habits of this population, combined with the international evidence on the risks associated with school environments, limits the understanding of how specific contextual factors—such as school schedule, availability of unhealthy foods in cafeterias, and the use of pocket money—impact diet quality and nutritional status in this less studied social stratum. Given this panorama, this study aims to quantitatively evaluate the consumption of macronutrients (total carbohydrates and fats) and critical micronutrients (sodium) in the diet of children aged 7 to 10 years enrolled in private schools in the municipality of Cotia (São Paulo, Brazil), correlating the data with nutritional status (classified by BMI), sex and, crucially, the school period (full‐time vs. part‐time).

## 2 Methods

### 2.1 Sample

A cross‐sectional, exploratory, and analytical study was conducted between January and August 2023 in two private schools in the city of Cotia (São Paulo, Brazil), a metropolitan region with a population density of 620.6 inhabitants/km^2^. The selected institutions represented contrasting profiles: School A, with 1400 students in part‐time (morning) classes, located in Granja Viana, and School B, with 350 students in full‐time courses, located in Parque Rincão.

The final sample consisted of 60 children aged 7–10 years (27 girls, 33 boys; mean age, 8.6 years), recruited by sending electronic questionnaires to caregivers, with response rates of 26.25% (42/160) and 30% (18/60) in schools A and B, respectively. Only questionnaires answered by caregivers who actively monitored the children's diet were included.

### 2.2 Food Evaluation

This study adopted a quantitative approach focused on nutrients that serve as critical markers of dietary quality and are most directly implicated in the health risks associated with the consumption of ultra‐processed foods (UPF), as defined by the NOVA (Martinez‐Steele et al. 2023) classification system (a food categorization system that focuses on the degree and purpose of industrial food processing rather than on nutrient content). Within the context of the rapid nutritional transition observed in Brazil, we prioritized the analysis of: sodium, a key indicator of food processing and a nutrient whose excessive intake is unequivocally linked to UPF; total carbohydrates, with particular attention to the quality of sources, given the high prevalence of free sugars and refined starches in these products; and total fats, examining the profile of consumption often skewed toward unhealthy saturated fats found in processed items. This focus is justified by public health data indicating that the excessive intake of these nutrients from UPF constitutes a more urgent and widespread issue in the studied demographic than protein inadequacy. The evaluation of water consumption was included due to its fundamental role in metabolic health and to identify patterns of hydration that may reveal substitution by sugary beverages, a common practice in urban populations with high access to processed foods.

To quantify the consumption of macro and micronutrients by children, a systematic approach based on digital platforms specialized in nutrition was adopted. Initially, the foods reported by the caregivers were converted into standardized measures (grams or milliliters) using equivalence tables from the YAZIO application, which provides the nutritional values per portion based on its extensive database. For example, an average banana (150 g) was associated with 0.00075 g of sodium, 0.5 g of total fat, 34.3 g of carbohydrates, and 112.4 mL of water, as recorded on the application. The data were then validated and refined through three additional platforms: Food Converter (2013–2025) (https://foodconverter.com/pt/), which compared volumetric measurements with equivalent weights in grams. Nutrium Software (https://nutrium.com/pt‐br): confirmed the nutritional composition of processed foods using information from labels and official databases. Diet Web Software (https://webdiet.com.br/): adjusted values for specific culinary preparations, considering variations in the cooking method.

For each child, the daily intake was calculated by multiplying the reported amount by the nutritional value per gram/mL of food. For example, if a child consumed two bananas (2150 g = 300 g), the total sodium ingested was 300 g (0.00075 g of sodium/150 g of banana) = 0.0015 g. This process was repeated for all 120 foods/beverages listed in the questionnaire, adding up the sodium, fat, carbohydrate, and water values at the end of the day.

Foods in nature may exhibit nutritional variations due to differences in maturation or origin. To minimize this, we used the average of the values available on the platforms. Foods such as snacks had their data extracted directly from the labels or manufacturers' websites when available.

The values were compared with the guidelines of the American Academy of Pediatrics (AAP) to ensure adherence to international standards (e.g., 2 g of sodium/day) (U.S. Department of Agriculture and U.S. Department of Health and Human Services 2020). Discrepancies between platforms were resolved by prioritizing information from official labels or consensus among at least three sources. A conversion matrix was created to standardize measures and avoid subjective interpretations.

The nutritional status was classified according to the WHO growth curves, categorizing children as eutrophic (percentile 5–85), overweight (85–95), or obese (> 95) (WHO 2006).

### 2.3 Water Intake Assessment

Total daily water intake (from water + other beverages + food moisture) was assessed against the recommended Adequate Intake (AI) for children aged 4–8 years (1.7 L/day) and 9–13 years (2.1 L/day for girls, 2.4 L/day for boys), as per the IOM range (Institute of Medicine (US) 2005). For analytical simplicity and due to the age distribution, the value of 1.75 L/day was adopted as a central reference point. Categories were defined as: below recommendation (< 1.75 L), optimal (≈1.75 L), and above recommendation (> 1.75 L).

### 2.4 Statistical Analysis

For statistical analysis, the Chi‐squared test was used to evaluate associations between adequate water/sodium consumption and variables such as nutritional status, sex, and school period. Differences in quantitative nutrient intake between groups (full‐time and part‐time) were assessed using the Mann–Whitney and Kruskal–Wallis tests, with significance set at *p* < 0.05.

## 3 Results

The demographic and nutritional profile of the studied sample showed that most children were male (55%), with an average age of 8.6 years. Regarding the distribution by school shift, students enrolled in part‐time or morning hours predominated (70%), while 30% attended full‐time hours. Regarding eating habits, it was found that almost all children had at least five meals a day, indicating an adequate feeding frequency in the studied population. The analysis of the relationship between nutritional status and school time revealed differences in the proportions of eutrophic children according to the study shift. There was a higher prevalence of eutrophication among students in the part/morning period (64.29%; *n* = 27) compared to those in the whole time period (44.44%; *n* = 8). In contrast, low weight was more frequent in full‐time (27.78%; *n* = 5) than in partial/morning (9.52%; *n* = 4). The overweight (16.67% vs. 16.67%) and obesity (11.11% vs. 9.52%) categories showed less pronounced variations between groups. Despite these numerical trends, the chi‐square test did not indicate statistical significance (*χ* ^2^ = 3.681; *p* = 0.2980), suggesting that the observed differences may be attributable to sampling variations. The total data highlight that 58.4% (*n* = 35) of the evaluated children were eutrophic, followed by 16.7% (*n* = 10) with overweight, 15% (*n* = 9) with low weight, and 10% (*n* = 6) with obesity.

The exclusive consumption of water (not including other liquids) identified that only 15% of the children (*n* = 9) reached the optimal daily intake (1750 L). In total, 51.7% (*n* = 31) of the children consumed water above recommendation level, 15% (*n* = 9) at the optimum level, and 33.4% (*n* = 20) below recommendation. When analyzing total daily liquid consumption (including water and other beverages), it was observed that 51.7% of children reached the optimal amount of water recommended. In comparison, nearly half of the participants (48.3%) remained below this recommendation.

The daily intake of sodium revealed that 38.3% of children (*n* = 23) consumed amounts exceeding the recommended limit (more than 5 g/day). The stratified distribution showed that only 13.3% (*n* = 8) reached the ideal intake (2 g/day), while 48.3% (*n* = 29) were in the borderline range (2–5 g/day), 25% (*n* = 15) in the worrying range (5–8.5 g/day), and 13.3% (*n* = 8) had excessive consumption (> 8.5 g/day). No child reached the recommended minimum intake for total fats, and the same was observed for carbohydrates. This finding, that all children consumed carbohydrates and total fats below the recommended levels, appears paradoxical given the high prevalence of overweight and obesity in this sample. To avoid misinterpretation, it is important to clarify that these values represent the absolute intake derived from a single 24‐h recall. Therefore, this discrepancy may be partly explained by the possibility of underreporting or misestimation by caregivers, which is a well‐recognized limitation of dietary assessment methods in pediatric populations. In Table 1, the 10 most consumed foods that are sources of sodium, carbohydrates, and total fats were listed.

**TABLE 1:** Main dietary sources of carbohydrates, sodium and total fats in the infant diet: Amount consumed, typical portion and contribution to daily intake.

Food	Total consumption (g)	Average per child (g)	Typical portion (g)	% of daily recommendation^a^
Carbohydrates				
Snacks and candies	4752	79.2	30 (1 pack)	28.8%
White rice	4057	67.6	100 (4 tablespoons)	24.6%
Simple cake	3600	60.0	60 (1 medium slice)	21.8%
Cheese bread	2236	37.3	50 (1 unit)	13.6%
Beans/lentils	2148	35.8	80 (1 ladle)	13.0%
French bread	2115	35.3	50 (1 unit)	12.8%
Bread form/syrium/toast	2037	33.9	50 (2 slices)	12.3%
Corn flour/cassava flour	1523	25.4	50 (2 tablespoons)	9.2%
Banana	1327	22.1	120 (1 unit)	8.0%
Baked savory snacks	1140	19.0	50 (1 unit)	6.9%
Sodium				
Cheese bread	41.6	0.7	50 (1 unit)	35.0%
Savory biscuit	36.9	0.6	30 (3–4 units)	30.0%
Simple cake	35.5	0.6	60 (1 slice)	30.0%
French bread	24.3	0.4	50 (1 unit)	20.0%
Bread/flatbread/toast	19.6	0.3	50 (2 slices)	15.0%
Homemade bread/sweet bread	18.0	0.3	50 (1 slice)	15.0%
Packaged snacks	12.7	0.2	30 (1 pack)	10.0%
Light bread (white or wholemeal)	12.1	0.2	50 (2 slices)	10.0%
Pork sausage	11.1	0.2	50 (1 unit)	10.0%
Green corn	10.2	0.2	80 (3 tablespoons)	10.0%
Total fat				
Beef—steak	1221.2	20.4	100 (1 small filet)	30.4%
Light bread (white or wholemeal)	1132.8	18.9	50 (2 slices)	28.2%
Cheese bread	1040.0	17.3	50 (1 unit)	25.8%
Simple cake	1015.3	16.9	60 (1 slice)	25.2%
Chicken breast—steak	946.4	15.8	100 (1 small filet)	23.6%
Packaged snacks	630.1	10.5	30 (1 pack)	15.7%
Walnuts/chestnut/pistachio/almonds	586.8	9.8	30 (1 handful)	14.6%
Pork sausage	524.8	8.7	50 (1 unit)	13.0%
Pork—steak	464.5	7.7	100 (1 small filet)	11.5%
Milk and derivatives	432.3	7.2	200 mL (1 glass)	10.7%

*Note:* The list shows the 10 foods that most contributed to the consumption of each nutrient in the total sample (*n* = 60 children).

^a^ Percentages were calculated based on a reference Value Caloric Total (VCT) of 2000 kcal, with carbohydrates set at 55% of VCT and total fats at 30% of VCT, in accordance with DRI recommendations. The sodium limit is 2000 mg/day per the WHO recommendation.

*Source:* Data collected through a 24‐h food reminder completed by parents/guardians.

The comparison of nutritional intake between different school periods is presented in Table 2. A significant association was observed between school schedule and carbohydrate consumption, with children enrolled full‐time presenting a markedly higher intake than their part‐time peers (*p* = 0.018). Furthermore, a trend toward higher sodium consumption was identified in the full‐time group, although it did not reach statistical significance (*p* = 0.073). No significant differences were found between the groups regarding the intake of total fats or any of the water consumption parameters (pure water, water from food, or total water).

**TABLE 2:** Intake of nutrients and water in children from 7 to 10 years, per school period.

Food components	School time		*p*
	Part‐time (*n* = 42)	Full‐time (*n* = 18)	
Sodium (g)	4.76 ± 2.7	6.9 ± 4.4	0.073
Total Fat (g)	181.3 ± 128.4	223.9 ± 147.6	0.154
Carbohydrates (g)	659.3 ± 259.9	873.4 ± 363.5	0.018*
Water in the food (mL)	2062 ± 1077	2222.7 ± 1397.2	0.493
Water (mL)	1083.3 ± 444.0	1138.9 ± 395.1	0.269
Total water (mL)	3145.5 ± 1001.2	3361.6 ± 1344.5	0.349

*Note:* Values expressed as mean ± standard deviation. Differences were tested by the Mann–Whitney *U* test. “Total Water” refers to the sum of “Pure Water” and “Food Water.”

* Statistically significant difference (*p* < 0.05).

The analysis of dietary intake according to nutritional status, detailed in Table 3, revealed a paradoxical pattern. Children classified as low weight exhibited the highest absolute intake of sodium, total fats, and carbohydrates. Notably, the mean sodium consumption in this group (6.5 g) was more than three times the maximum daily limit of 2.0 g recommended by the WHO. The post hoc analysis confirmed significant differences between the low‐weight group and the other nutritional status categories for most nutrients, underscoring that a higher absolute intake of these markers is not exclusive to children with overweight or obesity. This suggests that while caloric intake may be adequate or excessive, its quality is compromised across all groups, with the low‐weight children potentially consuming energy‐dense but nutrient‐poor foods.

**TABLE 3:** Nutrient and water intake in children aged 7–10 years by nutritional status: Multivariate analysis with Kruskal–Wallis test.

Food components	Low weight (LW) (*n* = 9)	Eutrophic (EU) (*n* = 35)	Overweight (OW) (*n* = 10)	Obesity (OB) (*n* = 6)	*p*
Sodium (g)	6.5 ± 4.9^a^	5.1 ± 3.2^b^	5.6 ± 3.2^b^	4.8 ± 2.2^b^	< 0.001*
Total fats (g)	241.7 ± 157.8^a^	189.2 ± 141.3^a,b^	197.3 ± 121.9^a,b^	146.1 ± 66.2^b^	< 0.001*
Carbohydrates (g)	812.6 ± 333.2^a^	704.9 ± 273.1^a,b^	773.9 ± 423.4^a,b^	614.2 ± 274.2^b^	< 0.001*
Water in the food (mL)	2353.4 ± 1461.7^a^	2114.3 ± 1092.4^a,b^	2221.6 ± 1434.6^a,b^	1536.5 ± 674.5^b^	< 0.001*
Water (mL)	1027.8 ± 475.1^a^	1021.4 ± 394.7^a^	1350.0 ± 394.4^b^	1250.0 ± 500.0^a,b^	< 0.001*
Total water (mL)	3381.2 ± 1219.7^a^	3135.8 ± 1058.3^a,b^	3571.6 ± 1309.9^a^	2786.5 ± 911.2^b^	< 0.001*

*Note:* Values represent absolute daily intake (mean ± standard deviation) for each nutrient and water, not relative adherence to dietary guidelines. The global comparison between the groups was performed using the Kruskal–Wallis test (significance level of 5%). Superscript letters (a, b) indicate the results of the post hoc Dunn test. Values sharing the same letter within a row do not differ significantly (*p* > 0.05). Values with different letters are statistically different (*p* < 0.05). “Total Water” refers to the sum of “Pure Water” and “Water in the Food.”

* Statistically significant difference in the overall comparison (*p* < 0.05).

## 4 Discussion

Despite the adequate feeding frequency (5 meals/day) observed in the studied population, it is essential to contextualize this finding in relation to other identified nutritional inadequacies, such as excessive sodium consumption, inadequate water intake, and energy imbalance. Multiple daily meals, although associated with metabolic benefits such as increased glycemic control and satiety, do not guarantee the nutritional quality of food choices (Pérez‐Escamilla 2023; Phetla et al. 2024). Specifically, the combination of high frequency with a diet rich in ultra‐processed foods can explain paradoxes, such as the coexistence of obesity with low intake of water or essential micronutrients (Louzada et al. 2021). This discrepancy suggests that dietary practices in schools or households may prioritize convenience and palatability over nutritional balance, even in strata with greater access to information (Cainelli et al. 2021).

The evaluation of total water consumption (pure water + liquids from other sources) revealed that 51.7% of children reached the recommended daily intake (1.75 L). However, almost half (48.3%) remained below the recommendation, even considering the contributions of water‐rich foods (such as fruits and soups) and beverages like juices and milk. The average consumption of pure water (1 L/day) corresponds to only 52.6% of the recommended volume, indicating a dependence on other liquid sources to compensate for the deficit. This pattern may mask risks, as sugary beverages (such as soft drinks and industrialized juices) are often incorporated into a child's diet and contribute to caloric excess and metabolic imbalances, even in children with adequate total fluid intake. Such findings reinforce the need for guidelines that prioritize not only the quantity but also the quality of liquids consumed, aligning with recommendations to encourage the consumption of pure water and reduce the intake of processed beverages (Ali et al. 2019).

High sodium consumption is a critical public health challenge in the Americas. The World Health Organization (WHO) recommends a maximum sodium intake of < 2 g/day (equivalent to < 5 g of table salt). However, regional consumption varies widely, ranging from 3.4 to 6 g of sodium per day (8.5–15 g of salt/day) (Organización Panamericana de la Salud 2021). The results of the present study reveal that more than half of the children (51.7%) exceeded this recommended daily limit for sodium. In our sample, this excessive intake is evidenced by the list of predominant food sources (Table 1), which is dominated by items such as cheese bread, savory biscuits, simple cakes, and packaged snacks—foods that are typical vectors of this low‐quality, high‐calorie dietary pattern.

A generalized deficit in the daily intake of total fats and carbohydrates was observed in our study, with all children falling below the established recommendations. This finding, apparently paradoxical in a sample where 26.7% were overweight or obese, can be interpreted as a key manifestation of the “double burden of malnutrition” at the individual level. This condition is characterized by “hidden hunger”—a diet sufficient or excessive in calories but deficient in essential nutrients and quality macronutrients, often resulting from the high consumption of ultra‐processed foods (UPFs) (Martinez‐Steele et al. 2023).

Although total fat intake was low, there are qualitative concerns. The low overall intake may mask the predominance of inappropriate sources, such as saturated and trans fats found in processed foods, which are linked to cardiometabolic risks even in small amounts. Concurrently, the deficit in carbohydrates was marked by a predominance of refined sources (e.g., sugars, white bread) over complex ones (e.g., whole grains, fruits), compromising fiber and micronutrient supply.

Thus, the quantitative deficit coexists with compromised nutritional quality. This scenario is exacerbated by the high availability of processed foods in school and home environments. The consumption of industrialized snacks and cakes, cited as primary sources (Table 1), reflects a diet based on rapidly absorbed energy but poor in structuring nutrients. Therefore, the observed deficits likely signal a severe qualitative imbalance, where processed calories displace nutrient‐dense foods rather than indicating a simple lack of food.

In Brazil, recent evidence observed in a study by Bratkowski et al. (2020) suggests an insufficient consumption of vegetables, associated with an increase in the intake of ultra‐processed foods, such as chips, biscuits, and industrially produced bread. This pattern aligns with the findings of this study. These habits, marked by high energy density, excess fat, and low fiber content, are recognized in the literature as risk factors for overweight and obesity. The Brazilian reality is not isolated; studies conducted in countries such as Mexico (Galvan‐Portillo et al. 2018), China (Zhen et al. 2018), and New Zealand (Saeedi et al. 2018) have also identified predominantly unhealthy infant eating patterns, characterized by a predominance of processed foods and nutritional poverty. These data reinforce the urgency of public policies that, as suggested by the results of this research, prioritize the reduction of ultra‐processed foods and encourage more balanced food choices and adequate hydration.

The findings of this study reveal an association between prolonged school routines and less healthy eating patterns, particularly concerning the consumption of refined carbohydrates and industrially processed foods. As shown in Table 2, children enrolled full‐time presented a significantly higher carbohydrate intake compared to those in part‐time (*p* = 0.018). This difference may be related to greater exposure to processed snacks, such as breads and cakes, which are commonly available in these environments. Although sodium consumption did not reach statistical significance (*p* = 0.073), the higher intake trend observed in the full‐time group is consistent with patterns of cumulative exposure to industrialized products, such as savory biscuits and packaged bread, aligning with recent evidence on the school food environment.

This dynamic is corroborated by previous studies, such as that of Silva, Marinho, et al. (2023), which, when evaluating the food offered in two schools in Macaé (Rio de Janeiro, Brazil), identified that the menus exceeded the recommendations of macronutrients established by current legislation. These results suggest that school supplies themselves may contribute to the excess of critical nutrients, such as simple carbohydrates, thereby reinforcing the hypothesis that a prolonged stay in school increases exposure to low‐quality nutrition.

In addition, Leite et al. (2021) demonstrated that the presence of school canteens is directly associated with increased consumption of ultra‐processed foods, including snacks, sugary drinks, and sweets, among adolescents. The study showed that the availability and variety of these products in canteens increases their consumption frequency. These data reinforce the influence of the institutional food environment on students' habits, especially in full‐time contexts where dependence on fast and industrialized snacks appears to be higher.

Early exposure to ultra‐processed foods was also observed by Silva, Traebert, et al. (2023), who identified frequent consumption of artificial juices (daily) and other industrialized food groups (1–2 times/week) in children from 6 years. These findings suggest that the consumption of processed products has become normalized since childhood, a pattern already documented in other age groups, as seen in the 2008–2009 Household Budget Survey (Henriques et al. 2021). The combination of this evidence suggests that a prolonged school routine, combined with lax regulations on food marketing in schools, may contribute to perpetuating cycles of poor nutrition, with implications for nutritional status and long‐term health.

Nutritional status also influenced eating habits, as observed in Table 3. Children with low weight consumed significantly higher amounts of sodium, fats, and carbohydrates compared to those with overweight and obesity (*p* < 0.05). This pattern may reflect strategies to achieve caloric needs, such as prioritizing energy‐dense but nutrient‐poor foods. In contrast, children with obesity presented lower sodium consumption, possibly due to dietary guidelines emphasizing the restriction of industrialized foods, which are commonly associated with weight control. This duality underscores that while absolute intake varies by nutritional status, the underlying issue of poor dietary quality—driven by ultra‐processed foods—persists across all groups.

Hydration patterns were also inversely related to nutritional status. As shown in Table 3, children with low weight and those classified as eutrophic consumed significantly less total water than children with obesity (*p* < 0.05). This finding may be explained by two non‐mutually exclusive hypotheses. First, specific recommendations for weight loss often include increased water intake, which could positively influence the hydration habits of children with obesity under dietary guidance. Second, the lower water consumption among eutrophic and low‐weight children may reflect a greater substitution of water by sweetened beverages, particularly in groups with higher access to caloric products. These results align with previous research indicating that targeted clinical interventions can modulate hydration behavior, while inadequate water intake is often associated with less healthy eating patterns.

In the study by St Pierre et al. (2022), the children with food insecurity, often associated with low weight, consume more processed foods rich in sodium and refined carbohydrates due to cost and limited access to fresh foods. The same was observed by Galvan‐Portillo et al. (2018), where children with food insecurity prioritize calorie‐dense foods (breads, pasta) to compensate for energy deficits.

Regarding hydration, Santos et al. (2022) identified, in a study with overweight patients in nutritional follow‐up, that those who underwent targeted clinical interventions (such as recommendations for increased water intake) had a significant increase in water consumption, reaching an average of 1.5 L/day. In contrast, eutrophic individuals of the same context maintained intake below 1 L/day, reinforcing the hypothesis that specific guidelines in weight management programs can positively modulate this behavior. This finding aligns with the dynamics observed in the present study, in which obese children and adolescents exhibited higher water consumption, which clinical guidelines may influence. On the other hand, Lucchesi et al. (Lucchesi et al. 2021) conducted a cross‐sectional analysis of adults from São Paulo, suggesting that lower water intake is associated with less healthy eating patterns, characterized by lower consumption of fruits, vegetables, and natural foods—groups that naturally contribute to hydration.

The interpretation of our findings must consider the study's contextual and methodological boundaries. The moderate sample size (*n* = 60), derived from two private schools, reflects the well‐documented challenge of conducting research in private educational settings, where administrative protocols and high parental demands can limit participation rates and study reach. This, coupled with a modest response rate to the questionnaires, affects the statistical power for some subgroup analyses and restricts the generalizability of the results to the broader population of Brazilian children in private education. Therefore, the observed trends, particularly the non‐significant association between school schedule and nutritional status (*p* = 0.298) and the pronounced differences in intake by nutritional status, should be interpreted as indicative patterns that warrant verification in larger, multicentric studies.

## 5 Conclusion

The results of this study revealed worrying dietary patterns, including excessive sodium and simple carbohydrate intake, critically associated with the full‐time school schedule. The analysis also identified an underconsumption of essential macronutrients, even among children with greater financial and educational access. This finding, indicative of the “double burden of malnutrition” at the individual level, suggests that contextual factors—such as restricted food preferences, family practices, and inadequate food provision in schools—compromise nutritional balance.

These findings challenge the assumption that greater financial resources automatically ensure a healthy diet, underscoring the need for structural interventions, such as regulating the food environment in private schools and implementing effective food education programs. Future research should investigate how psychosocial and cultural variables interact with school and family environments. Ultimately, public policies and school‐based programs must prioritize these actions to reverse this scenario and protect children's long‐term health.

## Author Contributions

**Daniel Ramos Olcerenko:** conceptualization; data analysis; investigation; writing – original draft, collected data, writing – review and wrote part of the article. **Bruna da Silva Miranda and Erica Vanessa Batista dos Santos:** the generation and data collection, collected data, performed most of the statistical analysis, and wrote part of the article. **Lucas Melo Neves:** writing – review and editing, final approval of the version to be published. **Patrícia Colombo‐Souza:** conceptualization; data analysis; investigation; writing – original draft; writing – review and editing; final approval of the version to be published.

## Funding

The authors have nothing to report.

## Ethics Statement

This study was conducted in accordance with the guidelines outlined in the Declaration of Helsinki, and all procedures involving research study participants were approved by the Ethics Committee of the University of Santo Amaro (CAEE: 67789023.9.0000.0081‐Opinion 5.943.541).

## Consent

Written informed consent was obtained from all subjects/patients.

## Conflicts of Interest

The authors declare no conflicts of interest.

## Data Availability

The data that support the findings of this study are available from the corresponding author, Patrícia Colombo‐Souza, upon reasonable request.
